# The impact of regular sperm donation on bulls’ seminal plasma hormonal profile and phantom response

**DOI:** 10.1038/s41598-021-90630-8

**Published:** 2021-05-27

**Authors:** Alicja Kowalczyk, Elżbieta Gałęska, Ewa Czerniawska-Piątkowska, Anna Szul, Leszek Hebda

**Affiliations:** 1 Department of Environment Hygiene and Animal Welfare, Wrocław University of Environmental and Life Sciences, Chełmońskiego 38C, 50-576 Wrocław, Poland; 2 Department of Ruminant Science, West Pomeranian University of Technology, ul. Klemensa Janickiego 29, 71-270 Szczecin, Poland; 3 Malopolska Biotechnic Centre Ltd, Krasne, Poland

**Keywords:** Reproductive biology, Animal behaviour

## Abstract

The aim of this study was to analyze the relationship between the concentration of hormones in the seminal plasma, the bull maintenance system in the insemination station, and the regularity of sperm donation and the response to the phantom (libido level). An additional goal was to determine whether there is a relationship between the hormonal profile in the blood, the sperm plasma, the oxidative and antioxidant profile in the blood of bulls and the biometry of their testicles and scrotum, as well as the quality of their sperm in both different seasons and intensities of reproductive use. For the study, 220 healthy and sexually mature Polish Holstein–Friesian bulls were used. They all had normal libido and were fed equally. The animals were grouped according to the scheme: young (16–20 month/n = 60) and old (26–30 month/n = 60) including: individually housed (n = 30) and group housed (n = 30) young, old individually housed (n = 30) and group housed (n = 30) (n total animals = 120); young animals donating semen once a week (every Thursday) (n = 25) and sporadically (once every two months on a random day of the week) (n = 25), old animals donating semen once a week (every Thursday) (n = 25 ) and sporadic donors (once every two months on a random day of the week) (n = 25) (n total animals = 100). When analyzing the results of this study, it should be stated that regular use has a positive effect on the secretion of sex hormones in bulls. Higher levels of testosterone and lower levels of estradiol and prostaglandins resulted in higher sexual performance, expressed by a stronger response to the phantom. The differences in favor of regular use were independent of the bull's age. The results of our research illustrate that the quality of semen and its freezing potential may depend on the season and frequency of its collection, as well as on the age of the males.

## Introduction

Insemination stations are losing income every day due to the growing number of males showing problems with sperm donation due to poor libido. Lack of semen means that the station will not produce insemination straws, and a bull showing symptoms of libido disorders is directed to slaughter, which generates even greater losses for the station. It has been observed that almost one quarter of breeding bulls maintained at bull stations are affected by poor libido^1^. The rush to introduce very young individuals into the production system occurred along with the dynamic development of the 'genomic era', which undoubtedly contributed to even greater problems with sexual immaturity in these animals. Moreover, there is a significant loss of time and an increased risk taken by technical personnel to stimulate bulls with a lower libido. Moreover, in such cases a much higher labor input is observed^2^. In the work of Bryant^3^ it was noted that libido is a response to endogenous or exogenous stimuli. This response may be mediated by various physiological mechanisms, male motivation and the experience acquired by the bulls. This parameter can be used to measure the reproductive competences of bulls^4^. This parameter is defined as the time elapsed from exposure to stimuli to the first service, and is calculated using the reaction time^5^. Ejaculation efficiency and semen quality are modified by the level of sexual arousal and performance^6–8^. High sex drive in bulls leads to better erection, penile protrusion, ejaculation thrust, libido, and female mating ability^9^.

Testosterone is the major androgen necessary for spermatogenesis in the testes and is mainly responsible for the maintenance of secondary sexual characteristics and libido and thus promotes sperm production in bulls^10^. Luteinizing hormone (LH) and follicle stimulating hormone (FSH) hormones are also responsible for male fertility. These hormones have the ability to modulate testosterone synthesis in Leydig cells. The pituitary-hypothalamus-gonadal axis regulates the secretion of GnRH, which is responsible for the secretion of gonadotrophins and testosterone needed for spermatogenesis, sperm maturation and reproductive behavior. In addition, adiponectin, which is a pleiotryopic regulator of many biological functions, may increase testosterone production, which contributes to an increase in libido. Adiponectin plays an important role in the structure and function of sperm and may also be involved in the fusion of the sperm with the oocyte^11^. Estrogen is the end product of the irreversible transformation of androgens by the aromatase enzyme. Although the influence of seasonality on sperm characteristics and male libido has been extensively studied in various breeds^12–15^, there are no reports on the influence of the intensity of reproductive use of bulls kept in insemination stations on their libido level and the concentration of hormones in the semen plasma. In many studies, the relationship between testosterone in the seminal plasma and sperm quality and sperm motility has not been proven^16,17^. However, there is contradictory information on fertility and testorerone levels in bulls^16,17^. Jeved et al.^18^ found a relationship between higher concetrations of testosterone in semen plasma and bull semen quality including parameters such as pH, motility and sperm count. Moreover, in the case of low libido in bulls, it has been suggested that this is due to low testosterone levels^16,17^.

This study aimed to analyze the relationship between the concentration of hormones in the semen plasma, the bull maintenance system in the insemination station, and the regularity of sperm donation and the response to the phantom (libido level). An additional goal was to determine whether there is a relationship between the hormonal profile in the blood, in sperm plasma, oxidative and antioxidant profile in the blood of bulls and the biometry of their testicles and scrotum, as well as the quality of their sperm in different seasons and different intensities of reproductive use.

## Materials and methods

### Animals

The experiment was performed as part of routine activities during the current semen production in the reproductive station and did not require the approval of the ethics committee. These experiments were performed in the Breeding and Insemination Centre ‘MCB’ (Krasne, Poland).

For the study, 220 healthy, sexually mature and equally-fed Polish Holstein–Friesian bulls with normal libido were used. The animals were grouped according to the scheme: young (16–20 months/n = 60), old (26–30 months/n = 60); including: individually housed (n = 30) and group housed (n = 30) young, old individually housed (n = 30) and group housed (n = 30) (n total animals = 120); young animals donating semen once a week (every Thursday) (n = 25) and sporadically (once every two months on a random day of the week) (n = 25), old animals donating semen once a week (every Thursday) (n = 25 ) and sporadic donors (once every two months on a random day of the week) (n = 25) (n total animals = 100). The trial was conducted in two phases over summer (May-Oct), and winter (Nov—April) season. The entire experience was conducted over 2 years.

### Semen collection and hormone concentration analysis

Two ejaculates were collected from each bull at 7 a.m. using an artificial vagina and the standard phantom intended for cattle. The ejaculates were then centrifuged to separate seminal plasma from sperm cells and the supernatant was used for the hormonal assay. Commercial ELISA kits were used to evaluate the endocrine profile of seminal plasma. The analyzes were performed according to the manufacturer's instructions. Testosterone was determined in seminal plasma of crossbred cattle bulls by Bovine Testosterone (T) ELISA Kit (Cusabio Technology, LLC, USA). The estrogen was detected using a Bovine Estradiol ELISA kit (Cusabio Technology, LLC, USA). Prostaglandin E2 was determined using Prostaglandin E2 ELISA kit (My Biosource, USA).

### Assessment of the reaction to the phantom

To assess the reaction to the phantom, a two-point scale was adopted depending on the speed of crossing the yellow line applied to the floor at a distance of 1.5 m from the phantom; low libido > 3 s, high libido < 2.9 s. Only jumps on the phantom after crossing the indicated line by the bull ended with ejaculation were considered correct. Before starting the timing, each male has the opportunity to sniff the phantom and mount it for the same amount of time (‘warm-up exercise’).

### Blood  collection and assessment of hormone profiles

Blood samples were collected by puncture of the jugular vein into heparin tubes (20 IU heparin per ml of blood) at four-week intervals at various times of the year. Blood samples were collected on the same day and at the same time from all animals.

Blood samples were centrifuged at 1200*g* for 15 min at 4 °C. Serum samples were separated, removed by pipette, labeled and stored at − 20 °C in a freezer until further analysis.

#### Hormonal profile

Serum LH and FSH concentrations were determined by the dual antibody RIA method, while testosterone and estriadol concentrations were determined by the solid-phase RIA method as previously described by Dance et al.^19^ and Moura et al.^20^.

#### Antioxidant profile

The antioxidant status of the bulls' serum was determined by the analysis of reduced glutathione (GSH), urid acid (UA), total protein (TP) and albumin (ALB). The obtained values of the abovementioned parameters were expressed per liter of blood^21^.

#### Oxidation profile

The intensity of the oxidative damage to the proteins was established by determining the concentration of the protein carbonyl content (PCC) and for the intensity of the oxidative stress was analysed blood serum lipid peroxidation assessment (TBARS values).

The concentration of lipid peroxides was determined spectrophotometrically by measuring the concentration of malondialdehyde and other reactive products of thiobarbituric acid resulting from free radical peroxidation of unsaturated fatty acids at high temperature and low pH.

PCC content was determined spectrophotometrically using 2,4 dinitrophenyl hydrazine (DNPH) according to the method of Levine et al.^22^. An extinction coefficient of 21.0 m^−1^ cm^−1^ was used to calculate the carbonyl content per gram of protein.

### Quality profiles of semen production

The volume of the ejaculate was measured by reading the graduated tube and the concentration was calculated spectrophotometrically.

The fresh undiluted semen was then evaluated microscopically (Nikon E 200, China) for mass motility. Subsequently, the semen was extended with animal protein–free commercial BIOXcell® extender (IMV Technologies, L’aigle, France) to a final concentration of 120 × 106 spermatozoa/mL and rated in terms of motile sperm percentage, progressive motility and viability. Semen was automatically packed (Bloc Machine FIN, IS 4, France) into polyvinyl chloride (PVC) straws (0.25 mL) (Biovet, France) which were filled and equilibrated for 1.5 h at 4 °C. After equilibration, the straws were frozen in liquid nitrogen vapor using a computer controlled automatic freezer from 4 to − 15 °C at the rate of − 3 °C /min and from − 15 to − 80 °C at the rate of − 10 °C /min (IMV Technologies, France)^23^.

After reaching − 80 °C, semen straws were plunged into liquid nitrogen and packaged in plastic goblets for 24 h of storage in the liquid nitrogen container. The straws were thawed in a water bath at 38 °C for 20 s and were then examined to evaluate the quality after thawing.

#### Motility assessment

Mass motility was examined in 20 µL of semen which was placed on a prewarmed slide without any cover slip and analyzed under microscope (Nikon E 200, China) equipped with phase-contrast optics (100×)^24^. The mass motility was scored into four scales: + no motion, ++ free spermatozoa moving without forming any waves, +++ vigorous movement with moderately rapid waves, ++++ very rapidly moving waves^23^.

Total sperm motility and progressive movement were examined using a Sperm Class Analyzer (SCA, version 5.1, Microptic, Barcelona, Spain), a light microscope (Nikon Eclipse E200). Just prior to analysis, semen was diluted 1:10 in a warm (25 °C) physiological solution (sodium chlorate 0.9%). Then, 2 μL of the prepared sample was placed in a Leja 4 analysis chamber (Leja Products B.V., Holland) at a thickness of 20.0 μm. The slide was placed on a stage warmer (38 °C). A minimum of 500 cells were evaluated, and depending on sperm concentration, five analyses were performed per sample^23^.

#### Viability

The double stain SYBR-14 with propidium iodide (L-7011 LIVE/DEAD Sperm Viability Kit; Invitrogen, Molecular Probes, Barcelona, Spain) using flow cytometer was applied (CytoFlex Beckman Coulter, B3-R1-V0, China). For this purpose, 50 µL of thawed semen was measured (37 °C for 20 s) and 940 µL NaCl (0.9%) and 5 µL SYBR14 were added. The whole was thoroughly mixed and then incubated (36 °C for 10 min) without light access. Subsequently, 5 µL of PI was remixed and incubated for 3 min without light, followed by a test^23^.

### Testicular and scrotal biometrics

Testicular volume was estimated according to the method described by Love et al.^24^ by using the following formula for volume of an ellipsoid, i.e., 4/3πabc, where, c = length/2, a = thickness/2 and b = width/2.

Scrotal circumference was estimated with the method explained by the Society of Theriogenology^25^. The scrotum was measured with a caliper and a tape measure. The circumference of the scrotum was measured at its widest point with a taut measuring tape.

### Climatic variation

The biometeorological factors, relative ambient temperature and relative humidity values were received from the meteorology station Institute of Meteorology and Water Management. Airport Meteorological Station, located at close proximity of the experimental station for estimation of temperature humidity index (THI) (Table 1).

**Table 1:** Climatological data during the experimental period in the area where the bulls were kept (mean ± SD).

Seasons	Sunshine hours (h)	Maximum rainfall (mm)	Maximum temperature (°C)	Minimum temperature (°C)	Relative humidity (%)
Winter	2.34 ± 0.61	11.67 ± 9.75	2.68 ± 3.38	− 1.98 ± 2.76	83 ± 5.73
Spring	6.24 ± 1.15	13.17 ± 8.18	15.38 ± 6.76	4.77 ± 5.29	71.42 ± 5.29
Summer	8.38 ± 0.51	24.00 ± 7.77	26.02 ± 1.16	14.4 ± 0.96	73.5 ± 4.71
Autumn	4.59 ± 1.46	10.17 ± 5.08	15.60 ± 5.68	6.22 ± 3.31	82.75 ± 3.86

The biometeorological factors, relative ambient temperature and relative humidity values were received from the meteorology station Institute of Meteorology and Water Management. Airport Meteorological Station, located at close proximity of the experimental station for estimation of temperature humidity index (THI).

### Statistical analysis

Using the analysis of variance with the LSD test, the influence of such factors as bull's age, housing system, semen collection season, and frequency of collection on the concentration of hormones in the semen plasma were determined. Correlations were calculated between the frequency of sperm collecting and the strength of response to the phantom. Spearman correlations were calculated using the Corr SAS Enterprise Guide procedure. The significance of differences between the means was estimated using the T-test and Fisher's NIR (Least Significant Difference) test. Pearson's correlations were estimated for pairs of the analyzed random variables using the CORR Sas Enterprise Guide procedure (SAS/STAT 9.4, 2013).

#### Statistical model

- Y_ijklm_ = µ + s_i_ + wb_j_ + sb_k_ + ej_l_ + e_ijklm_.
- Y_ijklm_—analyzed feature.
- µ—expected value of the feature.
- s_i_—season of sperm collecting i = 1, 2
- wb_j_—bull age class, j = 1, 2
- sb_k_—housing system, k = 1, 2
- ej_l_—frequency of sperm collecting – l = reg, unreg.
- e_ijklm_—random error effect.

## Results

Table 2 shows the concentration of testosterone, estradiol, and prostaglandins depending on the season, age of the studied individuals, and regularity of their use.

**Table 2:** Testosterone, estriadol and prostaglandin concentrations depending on the season and age of the male subjects (mean ± SD).

Hormone profiles	Age groups	Winter season				Summer season			
		Regular mean ± SD	LSD young/old	Irregular mean ± SD	LSD young/old	Regular mean ± SD	LSD young/old	Irregular ean ± SD	LSD young/old
Testosterone ng/mL)	Young	8.98^A^ ± 0.71	*	8.02^B^ ± 0.99	d	8.98^A^ ± 0.99		8.1^B^ ± 0.93	*
	Old	9.64^A^ ± 0.98	*	8.21^B^ ± 0.78	f	8.89^A^ ± 1.05		7.52^B^ ± 1.01	*
Estradiol pg/mL)	Young	301.6^a^ ± 10.33	*	317.3^a^ ± 14.65	a	351.1^a^ ± 17.08	*	374.8^a^ ± 17.62	*
	Old	322.4^a^ ± 15.15	*	350.7^b^ ± 16.78	b	336.5^a^ ± 15.41	*	361.4^a^ ± 14.9	*
Prostaglandin E_2_ pg/mL)	Young	21.0^a^ ± 0.8		19.79^b^ ± 0.79		24.8^a^ ± 1.07		24.24^a^ ± 1.1	
	Old	22.0^a^ ± 0.99		20.2^b^ ± 0.83		23.82^a^ ± 0.93		23.06^a^ ± 0.91	

Explanation: different letters in the same lines differ significantly; a, b (*P* < 0.05), A, B (*P* < 0.01); *P < 0.05; **P < 0.01; *LSD* least significant difference.

There was a negative and statistically significant correlation (r = − 0.32) between the concentration of testosterone and estradiol in the bulls’ seminal plasma. The correlation coefficient between the concentration of testosterone and prostaglandins was 0.11, while the correlation between the concentration of estradiol and prostaglandins was negative but statistically insignificant (r = − 0.12). Pearson's correlation coefficients were calculated without division by the summer/winter production season. Highly significant differences (*P* < 0.01) were found in the testosterone concentration in the bulls’ seminal plasma used regularly and irregularly. These differences were highly significant (*P* < 0.01) regardless of the age of the bulls and the season of their use. This dependence is explained by a higher concentration of testosterone, and thus a higher libido, in substances used regularly. Considering the winter season—among the bulls used regularly, older animals showed a statistically higher concentration of testosterone (9.64 ng/mL) compared to younger animals (8.98 ng/mL) (C-D). Among the bulls used regularly, a significantly higher concentration of testosterone was also noted in older animals (8.21 ng/mL) compared to in younger siblings (8.02 ng/mL), the difference turned out to be statistically significant (e–f). Statistically, in the winter season a significantly higher testosterone concentration was observed in younger bulls, not used regularly (8.1 ng/mL) compared to the concentration of this hormone in older animals (7.52 ng/mL). In the winter season, the concentration of estradiol in the seminal plasma of bulls used regularly was lower than in bulls used in the irregular cycle, while for older bulls the concentration was statistically significantly higher (350.7 pg/mL) compared with irregularly used bulls (322.4 pg/mL). Comparing the age groups in terms of estradiol concentration, statistically significant differences were found between the estradiol concentration in the groups used regularly and in a variable manner. In the summer season, the concentration of estradiol was higher in bulls used irregularly and in younger animals, but these differences were statistically insignificant. Statistically, a significantly higher concentration of prostaglandins was recorded in the winter season in older animals that were used regularly. In the summer season, the concentration of prostaglandins was statistically higher in the seminal plasma of younger animals than in older animals, regardless of the regularity of sperm collection.

When analyzing the behavior of bulls before jumping onto the phantom (Table 3), their reactions were divided into two classes: weak and strong reactions. Bulls showing a weak and strong reaction to the phantom were compared, regardless of the season of use. In bulls used regularly, significantly higher testosterone concentrations were noted statistically, both in individuals showing a weaker and strong response to the phantom. The highest concentration of testosterone was recorded in bulls used regularly and with a strong reaction to the phantom (9.54 ng/mL), and the lowest in young bulls, used irregularly. Differences between weak and strong phantom responses were related to testosterone concentration, and these relationships were statistically highly significant (C-D, **) (*P* < 0.01). The concentration of estradiol was statistically, significantly higher in the group of bulls with a poor reaction to the phantom, both used regularly and variably.

**Table 3:** The strength of the response to the phantom depending on the regularity of use and the concentration of hormones in the seminal plasma (mean ± SD) in connection with relationship between the male housing system and the concentration of hormones in theirseminal plasma (mean ± SD).

Hormone profiles	Weak		Strong		Housing system	
	Response to the phantom		Response to the phantom			
	Donating Regular mean ± SD	Donating Irregular mean ± SD	Donating Regular mean ± SD	Donating Irregular mean ± SD	Individual mean ± SD	Group mean ± SD
Testosterone (ng/mL)	8.03^A^ ± 0.71	7.83^B^ ± 0.99	8.98^A^ ± 0.99	8.1^B^ ± 0.93	8.22^a^ ± 1.16	9.55^b^ ± 1.29
LSD weak/strong	**	**	**	**		
Estradiol (pg/mL)	301.6^a^ ± 10.33	317.3^b^ ± 14.65	351.1^a^ ± 17.08	374.8^b^ ± 17.62	333.10^a^ ± 11.84	349.92^a^ ± 13.97
LSD weak/strong	*	*	*	*		
Prostaglandin E_2_ (pg/mL)	21.0^a^ ± 0.8	19.79^b^ ± 0.79	24.8^a^ ± 1.07	24.24^a^ ± 1.1	22.76^a^ ± 1.1	21,07^a^ ± 1.53
LSD weak/strong	*	*	*	*		

Explanation: different letters in the same column differ significantly; a, b (*P* < 0.05), C, D (*P* < 0.01), *P < 0.05; **P < 0.01.

^**#**^Only animals that donate re gularly are included.

When the animals donated sperm regularly, males kept in the group pen showed a statistically higher concentration of testosterone in the semen plasma (9.55 ng/mL) compared to the animals kept individually (8.22 ng/mL). The concentration of estradiol in the plasma of semen of bulls housed regularly (housed in a single pen) (333.10 pg/mL) was lower than that of bulls housed in a group pen (349.92 pg/mL). Prostaglandin concentration was higher in bulls kept in a single pen (22.76 pg/mL) than in bulls kept in a group pen (21.07 pg/mL).

Statistically significant differences were observed (Table 4) between PCC and TBARS for extreme seasons (winter–summer), with the lowest PCC values (12.3 mmol/L and 16.3 mmol/L) recorded in summer and the highest in spring (17.2 mmol/L in young bulls). In older bulls, the concentration of PCC reached the highest value in winter (20.2 mmol/L). Differences between age groups regarding PCC content were statistically, highly significant (*P* < 0.01). Statistically significantly higher (*P* < 0.01) TBARS values were recorded for younger bulls in the spring-winter season (1.1 mmol/L and 1.2 mmol/L) compared to the summer season (0.5 mmol/L). Moreover, in older bulls, it was observed that the concentration of TBARS showed the opposite tendency of changes than in the case of young bulls, as in the winter-spring season it was the lowest (0.4 mmol/L), while in autumn and winter it increased (0.9 mmol/L and 1.0 mmol/L). There were no significant differences between the TBARS content in age groups for the summer-autumn seasons. The correlation between PCC and TBARS was 0.812 and was statistically, highly significant (*P* < 0.01).

**Table 4:** Concentrations of oxidative stress parameters in the blood serum of younger and older bulls in relation to different season (mean ± SD).

Season	PCC (mmol/L)		Test t	TBARS (mmol/L)		Test t
	Young	Old	Y/O	Young	Old	Y/O
Winter	16.3 ± 0.5^a^	20.2 ± 0.8^a^	**	1.1 ± 0.6^a^	0.4 ± 0.1^b^	*
Spring	17.2 ± 0.8^ab^	19.4 ± 0.7^ab^	**	1.2 ± 0.7^a^	0.4 ± 0.1^b^	*
Summer	12.3 ± 0.9^b^	16.3 ± 0.4^b^	**	0.5 ± 0.1^b^	1.0 ± 0.5^a^	ns
Autumn	16.1 ± 0.4^ab^	17.2 ± 0.3^ab^	**	0.7 ± 0.2^ab^	0.9 ± 0.3^ab^	ns
r	PCC					
TBARS	0.81 **					

Description: Values with different letters (a, b) differ significantly between rows at *P* < 0.05.

*PCC *protein carbonyl content, *TBARS* thiobarbituric acid reactive substrates.

Test T—t-Student’s test, **P ≤ 0.01; *P ≤ 0.05.

Analyzing the concentration of TP (Table 5) in the blood, it was found that it increased with warmer seasons. The difference between older and younger bulls was significant (*P* < 0.05) in the spring-winter seasons, and in the following seasons no significant differences between the age groups were found. In terms of ALB concentration, the results remained at a similar level throughout the study period, reaching the lowest values ​​in winter and spring, respectively (Y: 32.0; O: 34.4 g/L and Y: 33.2; O: 37.1 g/L), and the highest in summer and in the fall (Y: 36.1; O: 35.0 g/L and Y: 34.7; O: 34.5 g/L). The lowest AU values ​​in both bull age groups were observed in winter (Y: 40.3; O: 55.1 mmol/L), and the highest in summer (Y: 52.2; O: 63.6 mmol/L), whereby these differences were statistically significant (*P* < 0.05). A higher GSH value in terms of concentration was observed in older bulls compared to young bulls. The lowest concentration of GSH in both examined age groups was observed in summer, and in turn, the highest in spring (Y: 26.1; O: 28.6 mmol/L) (Table 6).

**Table 5:** Concentration of nonenzymatic antioxidants in the blood serum of younger and older bulls in relation to different season (mean ± SD).

Season	TP (g/L) Young	TP (g/L) Old	Test Y/O	ALB (g/L) Young	ALB (g/L) Old	Test Y/O	UA (mmol/L) Young	UA (mmol/L) Old	Test Y/O	GSH (mmol/L) Young	GSH (mmol/L) Old	Test Y/O
Winter	75.1 ± 3.1^a^	81.3 ± 2.1	*	32.0 ± 2.2	34.4 ± 3.3	*	40.3 ± 3.5^a^	55.1 ± 1.9^a^	*	24.4 ± 0.7	28.0 ± 0.9	*
Spring	79.3 ± 2.4^a^	83.2 ± 2.4	*	33.2 ± 2.3	37.1 ± 3.1	*	44.2 ± 3.3^a^	57.6 ± 2.1^a^	*	26.1 ± 0.6	28.6 ± 0.5	*
Summer	88.5 ± 2.2^b^	84.3 ± 2.6	Ns	36.1 ± 4.2	35.0 ± 1.6	ns	52.2 ± 4.1^b^	63.6 ± 4.4^b^	*	20.1 ± 0.4	26.3 ± 0.2	*
Autumn	86.4 ± 2.1^b^	85.1 ± 3.2	Ns	34.7 ± 4.0	34.5 ± 1.7	ns	49.0 ± 3.9^b^	61.3 ± 4.1^a^	*	22.2 ± 0.6	27.1 ± 0.2	*

Explanation: Values with different letters (a, b) differ significantly between rows at *P* < 0.05.

*TP* total protein, *ALB* *a*lbumins, *UA* uric acid, *GSH* reduced glutathione.

Test—t-Student’s test, **P ≤ 0.01; *P ≤ 0.05; ns = *P* > 0.05.

**Table 6:** Concentration of hormones in the blood serum of younger and older bulls in relation to different season (mean ± SD).

Season	LH (mIU/mL) Young	LH (mIU/mL) Old	TEST T Y/O	FSH (mIU/mL) Young	FSH (mIU/mL) Old	TEST T Y/O	Testosterone (ng/mL) Young	Testosterone (ng/mL) Old	TEST T Y/O	Estradiol (pg/mL) Young	Estradiol (pg/mL) Old	TEST T Y/O
Winter	3.5 ± 1.1^a^	3.0 ± 1.5^ab^	NS	26.11 ± 5.5^a^	29.18 ± 6.6^a^	NS	101.55 ± 1.3^ab^	94.55 ± 1.3^ab^	**	4.4 ± 0.2^b^	5.6 ± 0.4^b^	*
Spring	3.16 ± 1.2^ab^	3.10 ± 2.0^ab^	NS	22.0 ± 7.2^a^	25.34 ± 5.3^a^	NS	92.30 ± 1.1^ab^	100.76 ± 1.1^a^	**	8.2 ± 0.2^a^	10.8 ± 0.3^a^	*
Summer	2.66 ± 1.4^b^	3.98 ± 1.9^a^	*	9.04 ± 3.1^b^	14.68 ± 1.9^b^	*	139.24 ± 1.6^a^	156.90 ± 1.6^a^	**	5.1 ± 0.5^ab^	8.2 ± 0.4^ab^	*
Autumn	2.71 ± 1.2^b^	2.87 ± 1.7^b^	NS	13.13 ± 2.2^b^	13.90 ± 1.7^b^	NS	86.07 ± 1.5^b^	44.71 ± 1.5^b^	**	7.04 ± 0.7^ab^	9.1 ± 0.6^a^	*

Explanation: Values with different letters (a, b) differ significantly between rows *P* < 0.05.

Test—t-Student’s test, **P ≤ 0.01; *P ≤ 0.05; ns = *P* > 0.05.

Analyzing the concentration of luteinizing hormone (LH), it can be noticed that in the case of young animals these values ​​decreased in the period from winter to summer, then in autumn they increased slightly, while in the case of older animals a reverse trend was observed and the concentration of LH increased from winter to summer, to then be lowered in the fall. There were no statistically significant differences in the concentrations of luteinizing hormone (LH) between young and old animals in winter, spring and autumn. The values ​​of LH concentrations in summer were significantly different (*P* < 0.05) depending on the age of the animal and amounted to 2.66 mIU/mL for young bulls and 3.98 mIU/mL for old bulls, respectively. In the case of old animals, these values ​​were higher. The LH concentration in the summer season, amounting to 2.66 mIU/mL, in young animals was significantly lower (*P* < 0.05) compared to those of all ages in the spring and winter seasons. The lowest concentration of LH was in the summer season in young animals (2.66 mIU/mL), while the highest concentration was in old animals in the same period (3.98 mIU/mL).

When analyzing the values ​​of follicle stimulating hormone (FSH) concentrations, a downward trend can be noticed in the period from winter to autumn, except for young animals in the autumn period, because in their case the concentration increased compared to the previous season, i.e. in summer, and amounted to 13.13 mIU/mL. In any single study period, no significant differences (*P* < 0.05) were observed in the concentration values ​​between animals in terms of their age. The concentration of FSH in spring and winter was significantly higher (*P* < 0.05) in bulls of all ages compared to summer and autumn months and amounted to 26.11 ± 5.5 for young bulls and 29.18 mIU/mL for old specimens in winter. In the spring season concentration of FSH was on level 22.0 mIU/mL in young males and in old 25.34 mIU/mL. The lowest concentration of FSH was observed in young animals in summer (09.04 mIU/mL) and the highest in old animals in winter (29.18 mIU/mL).

Analyzing the testosterone concentration, an upward trend can be noticed in the older animals from winter to summer, followed by a decrease in the described values. There were no statistically significant differences in testosterone concentrations, both in young and old animals, between winter, spring and summer months. A highly significant statistical difference (*P* < 0.01) was demonstrated in the fall in relation to the remaining seasons of the year, while in the autumn months younger bulls had statistically insignificantly higher testosterone levels than old animals. The lowest value of testosterone concentration was observed in old individuals in the fall (44.71 ng/mL), while the highest value was in old bulls in the summer period (156.90 ng/mL).

In the case of estradiol levels, the age groups did not significantly differ statistically at specific times of the year. Significant differences (*P* < 0.05) were, however, observed in the winter months, when the estradiol concentration was lower in both age groups compared to the rest of the year. The lowest estradiol concentration was found in winter in young animals and it is equal to 4.4 pg/mL. The highest concentration of estradiol was obtained in the spring months in older animals and was 10.8 pg/mL.

Highly significant (*P* < 0.01) correlations (Table 7) were noted between PCC and TBARS and ALB, as well as between TP and UA. Significant correlations (*P* < 0.05) were found between: PCC and TP, TP and ALB, and ALB and UA. The other correlation coefficients were statistically insignificant. Highly significant (*P* < 0.01) and positive correlations were noted between blood testosterone level and blood LH level (0.36) and between LH and FSH (0.69). Highly significant (*P* < 0.01) but negative correlations were found between blood testosterone content and estradiol content (-0.41) and between estradiol and FSH (-0.25). The correlations between FSH and testosterone in the blood as well as estradiol and LU levels were not statistically significant. The values of the correlation between the content of hormones in the blood and analogous hormones in seminal plasma were highly significant (*P* < 0.01), positive and reached 0.98–0.999.

**Table 7:** Correlations between the season and bulls’ blood parameters.

	PCC	TP	ALB	UA	GSH	ALL	Estradiol	FSH	LH
TBARS	0.81**	0.028ns	0,171ns	0,069ns	0.163ns				
PCC	–	0.321*	0.485**	0.032ns	0.079ns				
TP		–	0.466*	0.512**	0.139ns				
ALB			–	0.361*	0.014ns				
UA				–	0.144ns				
Summer	PCC	TP	ALB	UA	GSH				
TBARS	0.72**	0.048ns	0.179ns	0.069ns	0.163ns				
PCC	–	0.465*	0.485**	0.032ns	0.429ns				
TP		–	0.466*	0.513**	0.155ns				
ALB			–	0.362*	0.036ns	Testosterone	− 0.41**	0.11ns	0.36**
UA				–	0.064ns	Estriadol		− 0.25**	− 0.023ns
winter	PCC	TP	ALB	UA	GSH	FSH			0.69**
TBARS	0.81**	0.003ns	0,182ns	0,079ns	0.187ns	winter	Estradiol	FSH	LH
PCC	–	0.465*	0.485**	0.036ns	0.041ns	Testosterone	− 0.39**	0.031ns	0.39**
TP		–	0.466*	0.512**	0.136ns	Estriadol		− 0.14**	− 0.033ns
ALB			–	0.361*	0.037ns	FSH			0.61**
UA				–	0.146ns	summer	Estradiol	FSH	LH
						Testosterone	− 0.43**	0.11ns	0.44**
						Estriadol		− 0.22**	− 0.096ns
						FSH			0.54**

Explanation: **P ≤ 0.01; *P ≤ 0.05; ns = *P* > 0.05.

Table 8 shows the parameters of semen quality, taking into account age groups (Young–Old) and groups used on a regular and irregular basis. The volume of ejaculate was higher in the group of older (6.5 mL) and regular used bulls (old 6.5 mL; young 4.2 mL), but these differences were not statistically significant. Statistically, sperm concentration was highly significantly (*P* < 0.01) highest in older bulls (2,831 × 106/mm3) and in bulls from both age groups, but used on a regular basis. The percentage of live sperm was higher in animals donating sperm regularly (in both age groups) by an average of 6.69% (young) and 8.54% (old). These differences were statistically, significantly (*P* < 0.05) higher in the group of bulls giving sperm regularly, regardless of the age of the male. The opposite tendency was observed for the percentage of dead sperm—in the group of regularly used there were significantly less (*P* < 0.05) dead sperm than in the group that donated irregularly. There was no significant difference between age groups in this parameter. Regularly used bulls, regardless of age, had better sperm motility results (total motility and progressive movement). Moreover, the semen of older bulls showed better total motility and progressive movement in comparison to younger bulls, regardless of the regularity of semen collection. The mobility of the sperm mass was higher (+++) in older bulls used regularly than in younger males (++). There were no differences in sperm mass motility in bulls used irregularly.

**Table 8:** Semen quality parameters of young and old bulls during regular and irregular sperm donation (mean ± SD).

Parameters	Regular semen donation		LSD R/I Y	Irregular semen donation		LSD R/I O
	Young	Old		Young	Old	
Volume (mL)	4.2 ± 2.1	6.5 ± 3.2		3.9 ± 2.2	5.7 ± 3.1	
Concentration (× 10^6^/mm^3^)	1774 ± 221.6^A^	2831 ± 604.9^B^	**	1905 ± 373.62^A^	3004 ± 825.25^B^	**
Live (%)	83.52 ± 3.70	84.45 ± 4.53	*	76.83 ± 3.99	75.97 ± 5.12	*
Dead (%)	10.03 ± 1.11	9.90 ± 1.19	*	11.10 ± 2.72	11.75 ± 3.57	*
Total motility (%)	78.28 ± 5.36	80.02 ± 5.03	*	73.51 ± 6.79	75.25 ± 4.04	*
Progressive movement (%)	46.67 ± 0.88	51.16 ± 1.96	*	41.32 ± 1.23	44.19 ± 2.91	**
Mass motility	++	+++		++	++	

Explanation: Values with different letters (a, b) differ significantly between columns at *P* < 0.05, *P < 0.05; **P < 0.01.

Table 9 presents the values ​​of the traits related to the quality of fresh and frozen semen in bulls from two age groups and divided into seasons. In the winter season, fresh semen obtained from older males had a significantly (*P* < 0.05) higher volume (7.1 mL) and concentration (2598 × 10^6^/mm^3^) compared to young bulls (4.9 mL and 1646 × 10^6^/mm^3^). In terms of the mobility and viability parameters of fresh and frozen semen, no significant differences were observed during the winter season within the studied age groups. In contrast, in the spring season, both the volume of fresh ejaculate, its concentration and the percentage of live and motile sperm were significantly (*P* < 0.05) higher in the group of older bulls. The frozen semen of the younger bulls was statistically of significantly (*P* < 0.05) lower quality in terms of viability than the older bulls and contained on average 8.04% less viable sperm than the frozen semen of older males. In the summer season, ejaculate volume and sperm concentration differed significantly between young and old bulls, 1.2 mL and 962 × 10^6^/mm^3^ sperm, respectively. Moreover, in terms of sperm concentration, it was observed that in the summer season this value was the lowest among all analyzed seasons of use. Similarly, in terms of viability and sperm motility, the analyzed ejaculates were of the poorest quality in the summer season. However, in the autumn season, significant differences in terms of sperm mobility and viability were observed between older and younger males by analyzing their frozen semen. Older bulls were characterized by significantly (*P* < 0.05) better parameters of frozen semen than younger males. The highest mobility of sperm mass was observed in the winter and spring seasons.

**Table 9:** Quality parameters of fresh and frozen semen of young and old bulls depending on the season (mean ± SD).

Parameters	Fresh		Frozen		Season
	Young	Old	Young	Old	
Volume (mL)	4.9 ± 1.2^b^	7.1 ± 2.7^a^	–	–	Winter
Concentration (× 10^6^/mm^3^)	1646 ± 254.1^b^	2598 ± 555.76^a^	–	–	
Live (%)	79.45 ± 4.4	84.87 ± 4.2	53.50 ± 2.24	59.02 ± 4.57	
Dead (%)	13.33 ± 1.08	10.96 ± 0.97	42.13 ± 3.12	38.28 ± 3.99	
Total motility (%)	76.71 ± 4.89	80.81 ± 5.05	48.54 ± 3.65	52.10 ± 4.06	
Progressive movement (%)	41.30 ± 2.06	49.93 ± 5.15	30.30 ± 1.91	32.71 ± 2.04	
Mass motility	+++	+++	–	–	
Volume (mL)	3.9 ± 1.9 ^b^	5.1 ± 3.2^a^	–	–	Spring
Concentration (× 10^6^/mm^3^)	1247 ± 431.5^b^	2854 ± 477.65^a^	–	–	
Live (%)	72.15 ± 3.54^b^	86.70 ± 5.1^a^	49.59 ± 2.1^b^	57.63 ± 3.12^a^	
Dead (%)	11.44 ± 2.00	10.11 ± 1.16	50.08 ± 2.2^a^	40.06 ± 3.0^b^	
Total motility (%)	72.25 ± 3.93^b^	79.64 ± 5.47^a^	44.19 ± 3.26	49.77 ± 3.92	
Progressive movement (%)	44.42 ± 1.54	50.03 ± 4.98	31.14 ± 1.99	35.01 ± 1.96	
Mass motility	+++	+++	–	–	
Volume (mL)	3.8 ± 1.6^b^	5.0 ± 2.3^a^	–	–	Summer
Concentration (× 10^6^/mm^3^)	1151 ± 166.82^b^	2113 ± 453.12^a^	–	–	
Live (%)	67.29 ± 3.72	72.15 ± 3.91	44.05 ± 5.92^b^	54.67 ± 4.14^a^	
Dead (%)	16.17 ± 1.10	12.21 ± 1.01	51.99 ± 4.33	50.50 ± 5.18	
Total motility (%)	65.33 ± 3.28	67.90 ± 3.12	39.86 ± 3.77	42.94 ± 3.09	
Progressive movement (%)	36.44 ± 4.27	39.85 ± 4.66	28.88 ± 1.06	32.26 ± 1.13	
Mass motility	+ +	+ +	–	–	
Volume (mL)	4.1 ± 0.8^b^	5.2 ± 2.9^a^	–	–	Autumn
Concentration (× 10^6^/mm^3^)	1328 ± 205.68^b^	2355 ± 399.91^a^	–	–	
Live (%)	70.70 ± 2.92	75. 51 ± 4.16	46.01 ± 1.98^b^	56.30 ± 4.10^a^	
Dead (%)	13.06 ± 1.96	12.57 ± 1.10	44.99 ± 1.20	42.53 ± 2.28	
Total motility (%)	69.92 ± 4.05	73.12 ± 6.14	40.36 ± 2.70^b^	49.69 ± 4.44^a^	
Progressive movement (%)	37.00 ± 3.60	41.17 ± 4.62	30.11 ± 2.94^b^	37.71 ± 2.73^a^	
Mass motility	++	+++	–	–	

Description: Values with different letters (a, b) differ significantly between columns at *P* < 0.05

Table 10 resents the values ​​of the correlation along with their significance between the analyzed hormones, the volume and concentration of sperm, and the temperature on the day of sperm collection.

**Table 10:** Correlations of seminal hormone levels, semen volume and concentration, taking into account the semen collection season.

	Estradiol	Prostaglandin	Seminal volune	Concentration
**ALL**				
Testosterone	− 0.32**	0.11**	0.33**	0.31*
Estriadol		− 0.12**	0.04	− 0.10*
Prostaglandin			0.12*	0.28*
**Winter**				
Testosterone	− 0.28**	0.10**	0.35**	0.32*
Estriadol		− 0.13**	− 0.22	− 0.12*
Prostaglandin			0.13*	0.27*
**Summer**				
Testosterone	− 0.34**	0.14**	0.32**	0.29*
Estriadol		− 0.11**	− 0.11	− 0.11*
Prostaglandin			0.12*	0.26*

Explanation: **P ≤ 0.01; *P ≤ 0.05; ns = *P* > 0.05.

The testosterone level was negatively correlated with the level of estradiol (− 0.32) and positively with prostaglandin (0.11). The level of estradiol was also negatively correlated with the level of prostaglandins in the blood (− 0.12). The quoted correlations turned out to be statistically highly significant (*P* < 0.01). In the winter season, negative and highly significant correlations were noted between the levels of estradiol and testosterone (-0.28) and estradiol and prostaglandins (− 0.13). A similar tendency was observed in the summer season.

The volume of semen was positively and highly significantly correlated with the level of testosterone. Testosterone was positively and statistically significantly correlated with the value of sperm concentration. There were no significant correlations between the level of estradiol and the volume of semen, while the level of sperm concentration was positively correlated with the content of prostaglandins, and negatively with the content of estradiol in the blood. The above-mentioned correlations turned out to be statistically significant.

The testosterone level was negatively correlated (Table 11) with the level of estradiol (− 0.32) and positively with prostaglandin (0.11). The level of estradiol was also negatively correlated with the level of prostaglandins in the blood (− 0.12). The quoted correlations turned out to be statistically highly significant (*P* < 0.01). In the winter season, negative and highly significant correlations were noted between the levels of estradiol and testosterone (− 0.28), and estradiol and prostaglandins (− 0.13). A similar tendency was observed in the summer season.

**Table 11:** Correlations of hormone levels and semen quality, taking into account the semen collection season.

	Estradiol	Prostaglandin	Seminal volume	Concentration	Scrotal circumference (cm)	Testicular volume (cm^3)^	Total motility (%)	Dead (%)	Live (%)	Progressive movement (%)
**ALL**										
Testosterone	− 0.32**	0.11**	0.73**	0.31*	0.82**	0.72**	0.86**	− 0.88**	0.86**	0.88**
Estradiol		− 0.12**	0.04	− 0.10*	− 0.42**	− 0.34**	− 0.21*	0.32*	− 0.23*	− 0.41*
Prostaglandin			0.19*	0.28*	0.33**	0.24*	0.22*	0.12*	0.14*	0.21*
**Winter**										
Testosterone	− 0.28**	0.10**	0.35**	0.32*	0.79**	0.71**	0.82**	− 0.85**	0.82**	0.84**
Estradiol		− 0.13**	− 0.22	− 0.12*	− 0.42**	− 0.25**	− 0.19*	0.31*	− 0.22*	− 0.38*
Prostaglandin			0.13*	0.27*	0.31**	0.22*	0.18*	0.11*	0.13*	0.24*
**Summer**										
Testosterone	− 0.34**	0.14**	0.32**	0.29*	0.84**	0.73**	0.88**	− 0.89**	0.86**	0.92**
Estradiol		− 0.11**	− 0.11	− 0.11*	− 0.42**	− 0.34**	− 0.21*	0.32*	− 0.31*	− 0.44*
Prostaglandin			0.12*	0.26*	0.33**	0.24*	0.22*	0.11*	0.14*	0.19*
**Regular**										
Testosterone	− 0.31**	0.15**	0.42**	0.39*	0.82**	0.80**	0.85**	− 0.86**	0.83**	0.85**
Estradiol		− 0.22**	− 0.29	− 0.31*	− 0.53**	− 0.35**	− 0.39*	0.34*	− 0.32*	− 0.39*
Prostaglandin			0.14*	0.29*	0.34**	0.22*	0.21*	0.30*	0.23	0.24*
**Irregular**										
Testosterone	− 0.31**	0.14**	0.36**	0.29*	0.84**	0.73**	0.88**	− 0.89**	0.86**	0.92**
Estradiol		− 0.10**	− 0.13	− 0.11*	− 0.42**	− 0.34**	− 0.21*	0.32*	− 0.31*	− 0.44*
Prostaglandin			0.11*	0.26*	0.33**	0.24*	0.22*	0.11*	0.14*	0.19*

Explanation: **P ≤ 0.01; *P ≤ 0.05; ns = *P* > 0.05.

The values ​​of the correlation coefficients between the level of hormones and fresh semen quality parameters were determined. The volume of semen was positively and highly significantly correlated with the level of testosterone. Testosterone was positively and statistically significantly correlated with the value of sperm concentration. There were no significant correlations between the level of estradiol and the volume of semen, while the level of sperm concentration was positively correlated with the content of prostaglandins and negatively with the content of estradiol in the blood. The above-mentioned correlations turned out to be statistically significant (*P* < 0.05).

The testosterone level was correlated with the number of live spermatozoa, their motility and progressive movement highly significantly, with r exceeding 0.86, and negatively with the number of dead sperm (− 0.88). The level of estradiol was negatively correlated with the motility, progressive movement and live spermatozoa, and positively correlated with the level of dead sperm. Positive and statistically significant correlations were found between the analyzed characteristics of sperm quality and prostaglandins.

The testosterone level was highly significantly correlated with the area (0.82) and volume of the testicles (0.72). Similarly, highly significant, albeit negative, correlations were noted for the level of estradiol by area (− 0.42) and testicular volume (− 0.34). The prostaglandin level was positively correlated with the surface area (0.34) and testicular volume (0.24).

After applying the division according to the semen collection season and regularity, very similar correlation results between the analyzed values ​​were obtained.

In older bulls (Table 12), The circumference of the scrotum was greater by about 4 cm, both in the group of males used regularly and irregularly. The differences in the circumference of the scrotum were statistically insignificant. However, statistically significant (*P* < 0.05) differences in testicular volume were noted. Older bulls that regularly donate semen had a statistically significantly (*P* < 0.05) larger testicle volume by an average of 230.57 cm^3^ compared to younger males. This tendency was identical in the group of animals used irregularly, where the volume of testes in younger males was lower by an average of 564.34 cm^3^. Moreover, males used in an irregular manner were characterized by a greater circumference of the scrotum and a larger volume of testicles compared to males donating sperm regularly.

**Table 12:** Testicular biometry in young and old bulls during regular and irregular semen donation (mean ± SD).

Parameters	Regular semen donation		LSD R/I Young	Irregular semen donation		LSD R/I Old
	Young	Old		Young	Old	
Scrotal circumference (cm)	32.38 ± 1.56	37.95 ± 1.83		35.31 ± 2.01	39.22 ± 2.77	
Testicular volume (cm^3)^	991.05 ± 30.30^b^	1221.62 ± 38.26^a^	*	1114.57 ± 41.12^b^	1678.91 ± 69.84^a^	*

Explanation: Values with different letters (a, b) differ significantly between columns at *P* < 0.05, *P < 0.05; **P < 0.01.

## Discussion

The poor libido of bulls used in insemination stations is the main source of several difficulties related not only to the handling of such animals, their delayed inclusion in the current production of insemination doses, but most of all the generation of great financial losses. This is because bulls whose reproductive use does not bring profit due to their disorders of sexual encounters, are slaughtered^1^.

The determination of hormones, and in particular of reproductive hormones in semen plasma, is an important diagnostic and therapeutic marker of male fertility disorders.

Testosterone plays a key role in the expression of normal sexual patterns in bulls^10,18^, while the testosterone to estradiol ratio is important in their libido^26^. In our study, we found that regular use of bulls was associated with testosterone levels in semen plasma. Males that were used irregularly had a lower level of the hormone than bulls that regularly ejaculated. Moreover, bulls with higher testosterone levels reacted faster, more effectively, and more strongly to the phantom, which in turn allows us to hypothesize that regular sperm donation may improve bulls’ libido levels. Furthermore, we showed that the male group maintenance system was associated with higher levels of testosterone in their semen plasma, suggesting that this system promotes the expression of normal sexual patterns. Perhaps the higher level of this hormone is also due to the fact that during the contact of the bull with other animals, it can "train" the climbing reflexes of other males, which helps to maintain a proper level of libido.

We also showed that the concentration of estradiol was subject to seasonal fluctuations and was increasing with the passing of the winter season. Furthermore, the concentration of estradiol in the seminal plasma of bulls used regularly was lower than in bulls used in an irregular cycle. On the other hand, the bull's maintenance system was not related to the level of this hormone in their sperm plasma.

Prostaglandins present in the ejaculated semen acts through its receptors PTGER1 and PTGER3 and modulate sperm motility, capacitation, acrosome reaction, and fertilizing ability of spermatozoa^27^ by mediating an increase in intracellular calcium concentrations^28^. Moreover, increased concentration of this protein may lead to premature capacitation and acrosome reaction interfering with the fertilization process^29^. In this study, it was observed that the concentration of the above hormone in semen plasma was higher in the summer season than in the winter. Moreover, males used regularly were characterized by a higher concentration of this hormone in sperm plasma. There was also no relationship between the strength of the phantom response and the concentration of prostaglandins, similar to estradiol concentration.

We proved that bull age and regularity of their use were related to the biometric features of the testicles and scrotum. Similar results were obtained by Perumal et al.^30^, who reported that both the age of males and the season of sperm collection have an impact on the biometric features of male testes.

Analyzing the levels of hormones in the peripheral blood of bulls, we showed that they change with the season of the year. These data were also confirmed by other researchers in their experiments^30,31^.

Determining antioxidative and oxidative parameters in the serum of bulls may help in establishing the antioxidative and oxidative status of individual animals during different conditions such as season (temperature outside), housing system, regularity of sperm donating etc.

As the results of our research show, the quality of semen and its freezing potential may depend on the season and frequency of its collection, as well as on the age of the males. Balić et al.^32^ have shown that younger bulls are more sensitive to elevated ambient temperatures in summer, when increased pro-oxidative processes in semen plasma and sperm cause a decrease in sperm motility and, consequently, worsen sperm quality. Other researchers^33,34^ report that younger specimens achieve poorer sperm quality parameters than older ones, and that the season of the year significantly affects sperm mobility and viability. The results obtained by us also indicate that the older bulls had both a higher concentration of sperm in the fresh ejaculate, a greater volume of semen and a better viability and mobility (especially after freezing) compared to the young bulls.

Žaja et al.^35^ showed that bulls show high sensitivity to changes in climatic conditions in terms of the analyzed antioxidant and oxidation profile in the blood, which is also confirmed by the results of similar variability in the analyzed profiles.

## Conclusion

The conducted research shows that regular use has a positive effect on the secretion of sex hormones in bulls. Higher levels of testosterone and lower levels of estradiol and prostaglandins resulted in higher sexual performance, expressed by a stronger response to the phantom. The differences in favor of regular use were independent of the bull's age. As the results of our research show, the quality of semen and its freezing potential may depend on the season and frequency of its collection, as well as on the age of the males.

## Data Availability

The data underlying this article will be shared on reasonable request to the corresponding author.
